# An immunosuppressive macrophage profile attenuates the prognostic impact of CD20-positive B cells in human soft tissue sarcoma

**DOI:** 10.1007/s00262-019-02322-y

**Published:** 2019-03-16

**Authors:** Panagiotis Tsagozis, Martin Augsten, Yifan Zhang, Tian Li, Asle Hesla, Jonas Bergh, Felix Haglund, Nicholas P. Tobin, Monika Ehnman

**Affiliations:** 1 Department of Molecular Medicine and Surgery, Karolinska Institutet, Stockholm, Sweden; 2 Section of Orthopaedics, Karolinska University Hospital, Stockholm, Sweden; 3 Department of Oncology-Pathology, Karolinska Institutet, Stockholm, Sweden; 4 Present Address: Amcure GmbH, Eggenstein-Leopoldshafen, Germany; 5 Department of Oncology-Pathology, Karolinska Institutet, Radiumhemmet, Karolinska University Hospital, Stockholm, Sweden

**Keywords:** Sarcoma, CD20, MS4A1, IL10, M2 macrophages, Prognostic marker

## Abstract

**Background:**

Immune cells can regulate disease progression and response to treatment in multiple tumor types, but their activities in human soft tissue sarcoma are poorly characterized.

**Methods:**

Marker-defined immune cell subsets were characterized from a tumor microenvironmental perspective in two independent cohorts of human soft tissue sarcoma by multiplex IHC, quantitative PCR and/or bioinformatics.

**Results:**

B cell profiling revealed a prognostic role for CD20 protein (cohort 1, 33 patients) and MS4A1 gene expression (cohort 2, 265 patients). Multiplex IHC and gene correlation analysis supported a role in antigen presentation, immune cell differentiation and T cell activation. The prognostic role of MS4A1 expressing B cells was only observed in an IL10^low^, PTGS2^low^ or CD163^low^ tumor microenvironment according to the transcriptomic data. IL10 levels consistently correlated with the M2-like macrophage marker CD163, which also defined the majority of macrophages. A polarization of these cells toward a pro-tumoral phenotype was further supported by lack of correlation between CD163 and M1 markers like NOS2, as well as by low abundance of CD80 positive cells in tissue.

**Conclusions:**

Analysis of CD20/MS4A1 expression in soft tissue sarcoma merits further attention as a promising candidate prognostic tool for survival, but not in patients with a pronounced immunosuppressive tumor microenvironment. Macrophages are ubiquitous and polarized toward a protumoral phenotype. This provides a rationale for further studies on B cell function and immunotherapy targeting M2-polarized macrophages.

**Electronic supplementary material:**

The online version of this article (10.1007/s00262-019-02322-y) contains supplementary material, which is available to authorized users.

## Introduction

Solid neoplasms are composed of malignant cells and a stroma with non-neoplastic cells, where cells of the immune system are frequently abundant [1]. Immune cells typically have a prominent, but dichotomous role during tumor progression [2]. A primary function of the immune system is to combat malignancy. Yet, in the tumor microenvironment, immune cells often differentiate and gain tumor-promoting properties [3]. Anti-tumoral immune responses can however develop in patients with solid tumors and, importantly, be exploited therapeutically [4]. Thus, the balance between pro-tumoral and anti-tumoral activities plays a crucial role both in primary tumor growth and metastatic spread.

The significance of immune cells in soft tissue sarcoma (STS) is poorly characterized. These rare, heterogeneous tumors of mesenchymal origin have been the focus of only a few studies elaborating on the prognostic significance of selected immune cell populations [5–9]. Certain lymphocyte subsets have for example been associated with better prognosis. CD8-positive T cells are typically associated with direct anti-tumor functions through lysis of neoplastic cells, whereas T regulatory cells often suppress inflammatory responses in a context-dependent manner [10, 11]. In several tumor types, B lymphocytes are associated with good prognosis, but also the opposite has been reported, for example in renal cell cancer [12]. Macrophages are highly abundant in many tumors and can have supportive or inhibitory activities depending on the disease and type of treatment [13].

There is currently a great interest in targeting tumor-associated macrophages [14–16]. These highly diverse cells can likely regulate T cell activation and response to checkpoint-blockade immunotherapies in patients. They can also influence the effectiveness of chemo or radiation therapy [13]. Polarization of tumor-associated macrophages is intensely studied in tumor biology and the intricate balance between anti-tumoral activities of M1-like macrophages and pro-tumoral activities of M2-like macrophages is far from well understood [17–19]. Functional re-education, where the anti-tumoral activities of these cells can be triggered, is an interesting concept as an anticancer therapy, but therapeutic modulation of macrophage activity is likely to be complex and must be carefully evaluated in clinical trials [20, 21].

In the present study, we show that immune cells are abundant in the tumor microenvironment of STS. Immune cell heterogeneity and cell differentiation/polarization characteristics are specifically analyzed and related to cytokine gene expression, tumor microenvironmental properties and patient survival. Potential prognostic biomarkers are analyzed and reported according to the Reporting Recommendations for Tumor Marker Prognostic Studies (REMARK) Guidelines [22]. Three common B cell markers are particularly compared as candidate prognostic tools, whereby CD20 (gene name MS4A1) is identified as the preferred candidate in two independent STS cohorts.

## Materials and methods

### Patient inclusion and follow-up

The retrospective SARC TCGA sarcoma cohort contained 265 patient samples in total (see transcriptomic data below). Subtypes included 105 leiomyosarcoma, 58 dedifferentiated liposarcoma, 49 undifferentiated pleomorphic sarcoma/malignant fibrous histiocytoma/high-grade spindle cell sarcoma, 25 myxofibrosarcoma, 10 synovial sarcoma, 9 malignant peripheral nerve sheath tumor and 9 other types with frequencies less than 2%. Neoadjuvant radiotherapy and chemotherapy were exclusion criteria for the cohort [7]. The publicly archived dataset on sarcoma (TCGA, provisional) can be found at http://www.cbioportal.org/.

For the Karolinska STS cohort, 33 patients, 18 male and 15 female, with STS of the trunk or the extremities were prospectively included between 2013 and 2015. Patients were diagnosed with a high-grade STS through a standardized multidisciplinary approach at the Sarcoma Center Karolinska, Karolinska University Hospital [23]. Median age was 69 years (24–90). The most common histological diagnosis was undifferentiated pleomorphic sarcoma, present in 17 patients, whereas 7 patients were diagnosed with myxofibrosarcomas, 2 with angiosarcomas, 2 with malignant peripheral nerve sheath tumors, 2 with synovial sarcomas, 2 with malignant solitary fibrous tumors and 1 with leiomyosarcoma. Twenty-two tumors were deep seated, whereas 9 were subcutaneous. Twenty-two tumors were located in the lower extremities, 7 in the trunk or pelvis, and 4 in the upper extremities. Metastasis at diagnosis was present in one patient of the cohort. No patients received neoadjuvant radiotherapy or chemotherapy before tumor resection and collection of samples. Patient surveillance followed existing guidelines for high-grade STS [23]. Clinical examination and chest X-ray were done every 3 months for the first 2 years of follow-up, then bi-annually. Local recurrence and lung metastases were documented. Median follow-up was 48 months. Of the 33 patients, 23 were still alive at the last follow-up. Metastases were detected in seven patients during follow-up and local recurrence in two patients. Overall survival was 85% at 12 months and 66% at 36 months, whereas metastasis-free survival was 70% at 12 months and 57% at 36 months.

### Sample collection

Tissue samples for the Karolinska STS cohort were taken from macroscopically viable parts of the tumor (tumor periphery) and then formalin fixed and paraffin embedded or, alternatively, processed for RNA extraction. Typically, one sample of approximately 1 cm^3^ was taken from each tumor (median tumor size 244 cm^3^). Thirty tumors were available for immunostaining and 16 tumors were available for RNA extraction.

### Immunostaining

Formalin-fixed paraffin-embedded tumor sections with an average size of 0.8 cm^2^, and a thickness of 4 µm, were deparaffinized and rehydrated before heat-induced epitope retrieval at 110 °C for 5 min in a Decloaking NxGen Chamber TM (BioCare Medical). The unmasking buffer was selected according to the antibody product sheet recommendations with a preference for pH 6 (S2369, DAKO) when more than one buffer was listed. Sections were allowed to cool down for 30 min, equilibrated in TBS–Tween 20 (0.1%), and endogenous peroxidase activity was quenched by 3% H_2_O_2_ for 10 min if horseradish peroxidase-linked reagents were to be used. A 20-min incubation step with serum-free ready-to-use block (X0909, DAKO) was performed before applying the primary antibody. The antibodies used were directed against CD163 (NCL-L-CD163, Novocastra, 1:200), CD80 (MAB140, RnD, 20 µg/ml), CD8 (M7103, DAKO, 1:75), FOXP3 (ab20034, Abcam, 10 µg/ml, IHC), FOXP3 (12653, Cell Signaling, 1:100, immunofluorescence with liquid permanent red as below), CD68 (M0876, DAKO, 1:50), CD20 (M0755, DAKO, 1:200), CD19 (M7296, DAKO, 1:50) and PAX5 (12709, Cell Signaling, 1:100). Secondary detection reagents were chosen considering the species origin of the primary antibody and the type of enzyme label/visualization method preferred. For immunofluorescence, if not otherwise stated, Alexa Fluor 488 (A11001, Life Technologies, 1:300) and Alexa Fluor 594 (A11037, Life Technologies, 1:300) were used before mounting with Prolong diamond antifade mountant with DAPI (P36962, Life Technologies). For IHC, ImmPress reagent anti-mouse IgG, peroxidase (MP-7402, Vector laboratories), ImmPress reagent anti-rabbit IgG, peroxidase (MP-7401, Vector laboratories), ImmPress reagent anti-mouse IgG, alkaline phosphatase (MP-5402, Vector laboratories) or ImmPress reagent anti-rabbit IgG alkaline phosphatase (MP-5401, Vector laboratories) was used. Chromogenic substrates were DAB peroxidase substrate (SK-4100, Vector laboratories), liquid permanent red (K0640, DAKO), Vector blue alkaline phosphatase (SK-5300, Vector laboratories) or a combination of these according to colors shown in each image. Protocols for double labeling were optimized for sequential IHC with a second heat-induced epitope retrieval step at 80 °C for 5 min, followed by 20 min cooldown. If indicated in images, cell nuclei were counterstained with Mayer’s hematoxylin (01820, Histolab) before dehydration and mounting in permanent VectaMount mounting medium (H5000, Vector laboratories).

### Histoscoring of images

Each tissue section (approximately 0.8 cm^2^) was assigned an IHC score, where 0, 1, 2 and 3 indicated negative, low, moderate or high abundance, respectively, of each cell type. Stratification into two groups (low versus high) was done before performing survival analysis. Histoscoring was performed blinded to patient outcome (Monika Ehnman, PhD) and an independent observer (Yifan Zhang, MD, pathology resident) assisted with scoring and estimating the number of positive cells/section for B cell markers. The visual assessment was based on whole tissue section analysis and not hotspot analysis. Due to low cellular abundance and a strong trend toward prognostic significance in the first analysis, two additional sections from all tumor samples were immunostained for CD20. This resulted in an average tissue area of 2.4 cm^2^/tumor analyzed under the microscope in total. If no CD20 signal was detected in two out of three sections, the tumor obtained the lowest IHC score. Cohen’s kappa coefficient (*κ*) was calculated to measure inter-observer agreement (low/negative versus high/positive) for CD20.

### RNA extraction and quantitative real-time PCR

Tumor pieces were collected in RNAlater™ and RNA was subsequently isolated by Trizol followed by the GeneElute™ Mammalian Total RNA Miniprep Kit (Sigma-Aldrich) protocol including an on-column DNase digestion step. For cDNA synthesis, SuperScript II First-Strand Synthesis System for RT-PCR (Invitrogen) was used. SYBRgreen Universal PCR Master Mix (Applied Biosystems) was used in the PCR reaction with primers (Sigma-Aldrich) as follows: TTT GTC AAC TTG AGT CCC TTC AC (CD163, fwd), TCC CGC TAC ACT TGT TTT CAC (CD163, rev), ACG GCG CTG TCA TCG ATT (IL10, fwd), GGC ATT CTT CAC CTG CTC CA (IL10, rev), GCC CAG CAC TTC ACG CAT CAG (PTGS2, fwd), AGA CCA GGC ACC AGA CCA AAG ACC (PTGS2, rev), GCA GGT CGA GGA CTA TTT CTT TCA (NOS2, fwd), CGT AAG GAA ATA CAG CAC CAA AGA TA (NOS2, rev), CTC ATG GGC ACG GTG ATG (NOS3, fwd), ACC ACG TCA TAC TCA TCC ATA CAC (NOS3, rev), TGACACTGGCAAAACAATGCA (HPRT, fwd) and GGTCCTTTTCACCAGCAAGCT (HPRT, rev) with the latter primer pair used for normalization.

### Statistical analysis

Statistical analyses were done on pseudonymized data in the Karolinska STS cohort using SPSS software version 20. Overall survival (OS) was computed from the date of diagnosis to the date of last follow-up or death, and metastasis-free survival (MFS) from the date of diagnosis to the date of last follow-up or first distant metastasis. Survival analysis was per Kaplan–Meier, the parameters tested were dichotomized around the median, and the log-rank was used for comparison between groups. Hazard ratios between groups were calculated using a multivariate Cox regression analysis (proportional hazards model), where prognostic factors identified in the univariate survival analysis were balanced for sex and age. A Spearman rank test was used for analysis of immune cell marker correlations and correlations with standard prognostic markers. All tests were double sided, no *P* value correction was applied, and a *P* value of < 0.05 was considered significant.

For the SARC STS cohort transcriptomics, the *Z* score normalized gene expression values for MS4A1, CD19, IL10, PTGS2 and CD163 along with clinicopathological annotations were downloaded from cBioportal [24, 25]. Complete data were available for 258 patients in the SARC sarcoma dataset (TCGA, provisional). Gene expression values were split across all tumors into equal sized tertile groupings. All gene expression analyses were performed in R version 3.4.2 using the dplyr (version 0.4.1), survival (version 2.41-3) and survplot (version 0.0.7) packages. *P* values were adjusted for multiple testing using the Benjamini and Hochberg method with the *P.adjust function* of the R stats package version 3.5.0. The cBioportal software analysis tool identified mutually exclusive mRNA upregulations (none significant), and gene pairs with co-occurrent mRNA upregulations (2 significant) by Fisher’s exact test, and the results are presented with, and without, Bonferroni adjusted *P* values. All tests were double sided, and a *P* value of < 0.05 was considered significant.

## Results

### Tumor-associated macrophages outnumber lymphocyte subsets in STS

**Fig. 1:**
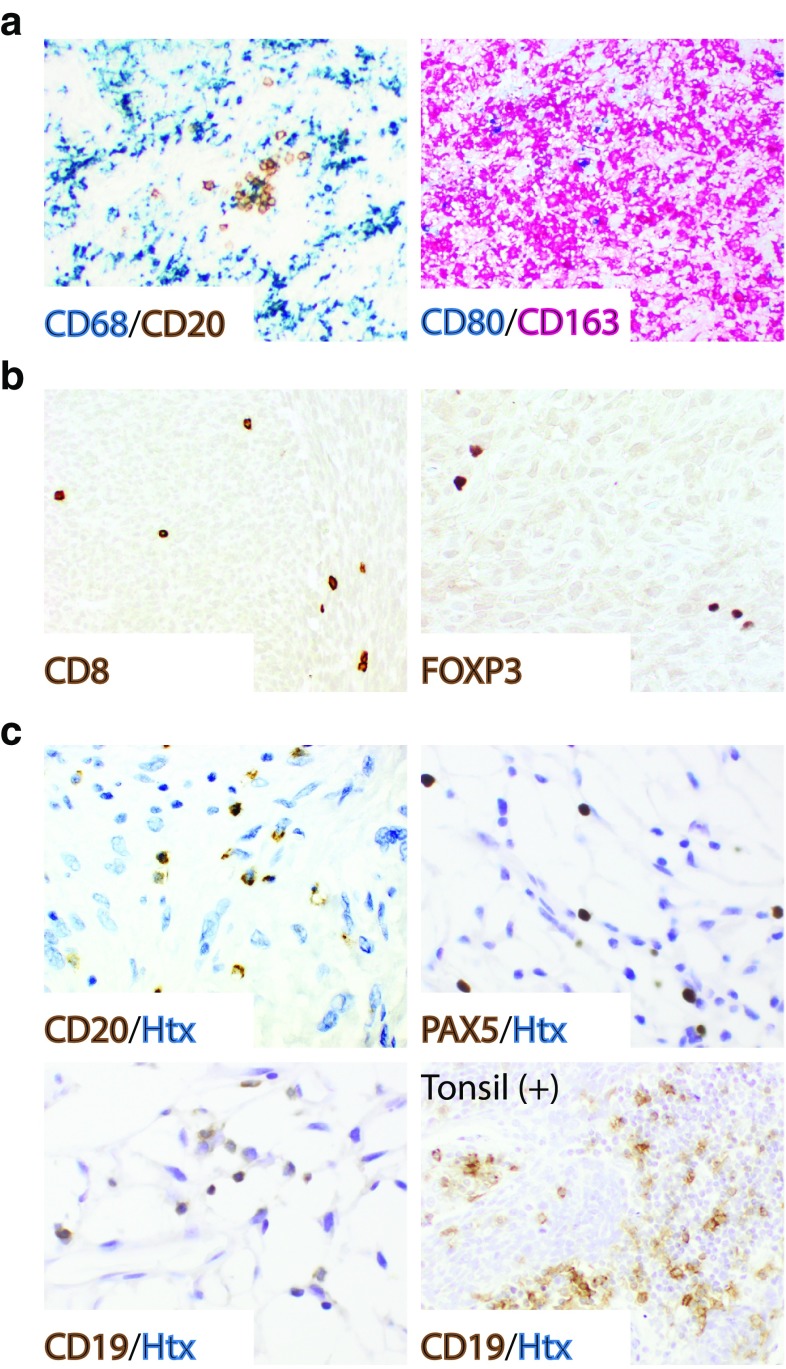
Tumor-associated macrophages outnumber lymphocyte subsets in STS. **a** Multiplex IHC using the pan-macrophage marker CD68 (blue, left), the B cell marker CD20 (brown, left), the M1-like associated marker CD80 (blue, right) and the M2-associated marker CD163 (red, right). **b** Immunostaining for CD8 (brown, left) and FOXP3 (brown, right). **c** Immunostaining for B cells using antibodies targeting CD20, PAX5 and CD19 (brown, Htx as counterstain, tonsil as positive control)

The presence of immune cell subsets was initially demonstrated by IHC of whole tissue sections from the Karolinska STS cohort. Three macrophage markers were used to detect tumor-associated macrophages: CD68, which is a pan-macrophage marker, CD163, which is considered to stain for tumor-associated macrophages polarized toward an M2 phenotype, and CD80, which is more associated with M1-like macrophages [26]. Infiltrating cells positive for CD68 and CD163 were detected in all tumors, and these cells were generally found at much higher density compared to lymphoid cell subsets such as CD20 (Fig. 1a). CD80-positive cells were only present in 9 out of 30 tumors, and at a low density. To compensate for these obvious differences in immune cell marker density, the histoscoring was adjusted in such a way that the highest IHC score for one marker could potentially correspond to the lowest score for another marker in terms of absolute cell number. The presented images illustrate the highest IHC score for each marker.

Infiltration of lymphoid cells was analyzed by immunostaining for CD8 (cytotoxic T cells), FOXP3 (T regulatory cells) and CD20, CD19 and PAX5 (B cells). CD8-positive cells were frequently detected (Fig. 1b), infiltrating all but one tumor, whereas FOXP3-positive cells were found in 16 out of 30 tumor sections. The CD20 staining was distinct (Fig. 1c), but sparse, and positive cells were only detected in 13 out of 30 investigated tumors (Supplementary Fig. 1a), and then, with approximately 40 cells/section found on average. Cohen’s kappa coefficient (*κ*) for inter-observer agreement (low/negative versus high/positive) was 0.87 for CD20, which supported very good agreement. PAX5 expression correlated well, but not completely, with CD20 expression (Supplementary Fig. 1a, b). The majority of tumors with PAX5-positive B cells only displayed 1–10 cells/section, which largely disqualified PAX5 as a useful marker. CD19 was almost undetectable using tonsil as a positive control.

### Correlation analysis demonstrates an M2-like macrophage phenotype

Correlation analysis showed that the pan-macrophage marker CD68 was associated with high immune cell infiltration in general, and with the other investigated myeloid markers, CD80 and CD163, in particular (Table 1). Given the high density of cells positive for the scavenger receptor CD163, RNA expression levels of other suggested M1/M2 macrophage polarization markers were analyzed. In this analysis, the M2 marker CD163 correlated with IL10 and PTGS2, but not with NOS2 and NOS3 (Table 2), supporting an M2-polarized phenotype.

**Table 1:** CD68 cellular density associates with other myeloid markers and high immune cell infiltration in general

	Immune cell markers							
	T cell subpopulations Macrophage subpopulations					B cell subpopulations		
	CD8	FOXP3	CD68	CD163	CD80	CD20	CD19	PAX5
CD8		0.695***	0.577**	0.612***	0.432*	0.377*	*0.034*	0.433*
FOXP3	0.000		0.455*	0.518**	*0.335*	*0.306*	*0.235*	*0.217*
CD68	0.001	0.012		0.712***	0.597***	*0.317*	*−0.274*	0.429*
CD163	0.000	0.003	0.000		0.440*	*0.180*	*−0.187*	*0.302*
CD80	0.017	0.070	0.000	0.015		0.574***	*0.024*	0.637***
CD20	0.040	0.101	0.088	0.342	0.002		*−0.095*	0.546**
CD19	0.858	0.211	0.143	0.323	0.899	0.792		*0.124*
PAX5	0.017	0.249	0.018	0.105	0.000	0.002	0.512	

Spearman rank test of IHC score correlations in the Karolinska STS cohort

*R* values in italics (upper right), *P* values (lower left) *P < 0.05, **P < 0.01, ***P < 0.001

**Table 2:** Tumor-associated macrophages are skewed toward an M2 phenotype

	Macrophage polarization phenotype				
	CD163	IL10	PTGS2	NOS2	NOS3
CD163		0.867***	0.700**	*0.214*	*0.493*
IL10	0.000		0.724**	*0.382*	*0.338*
PTGS2	0.005	0.002		*0.203*	*0.295*
NOS2	0.444	0.160	0.468		*0.193*
NOS3	0.062	0.218	0.286	0.430	

Spearman rank test of RNA expression correlations in the Karolinska STS cohort

*R *values in italics (upper right), *P* values (lower left) **P < 0.01, ***P < 0.001

### Presence of CD20-positive cells in whole tissue sections is prognostic for metastasis-free survival and overall survival

**Fig. 2:**
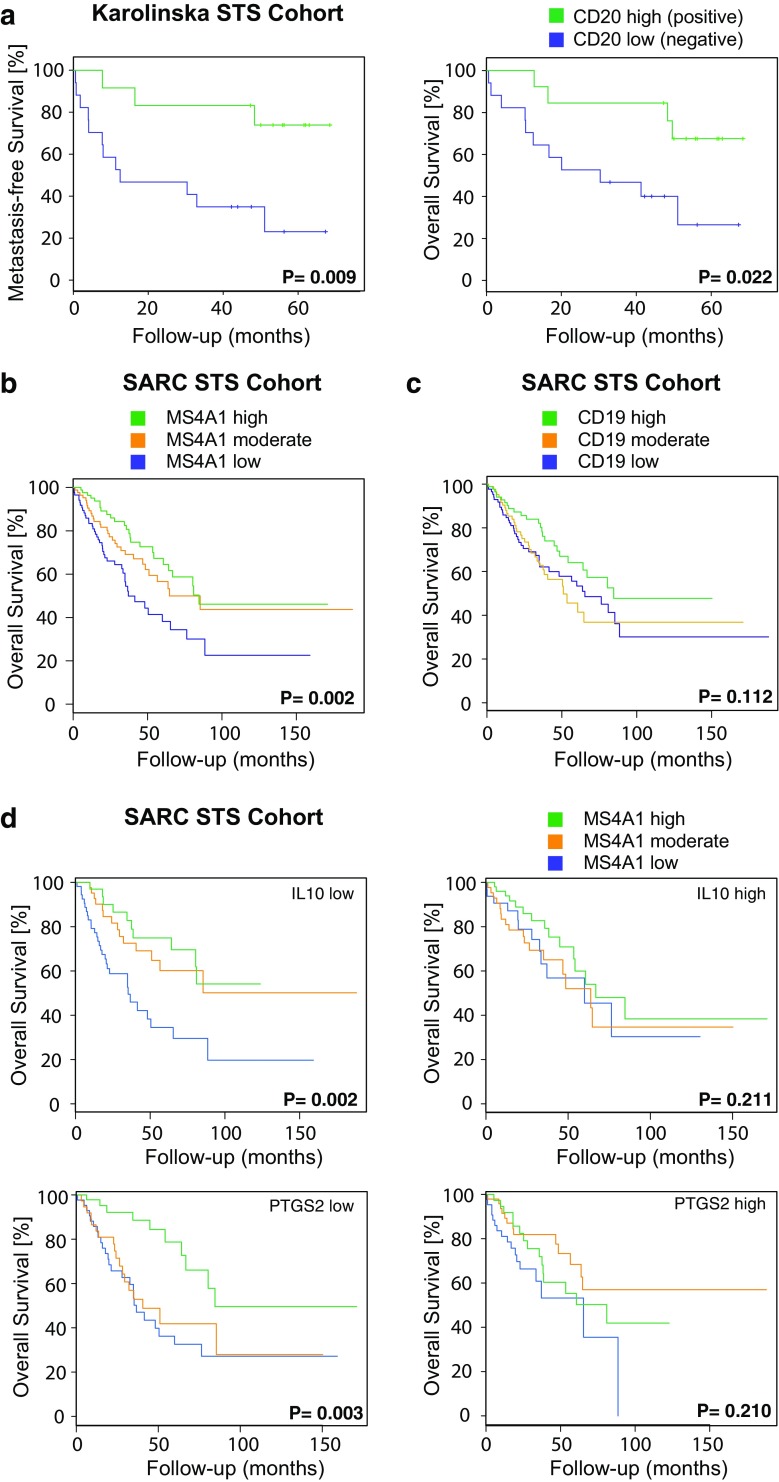
CD20/MS4A1 expression is prognostic, but only in IL10^low^ and PTGS2^low^ tumors. **a** Kaplan–Meier analysis illustrating the association between CD20 B cell-positive tumors and improved metastasis-free survival (left) and overall survival (right) in the Karolinska STS cohort. **b** Kaplan–Meier analysis illustrating the association between MS4A1 expression and improved overall survival in the SARC STS cohort. **c** Kaplan–Meier analysis of CD19 expression and overall survival in the SARC STS cohort (right). **d** Kaplan–Meier analyses illustrating the prognostic impact of MS4A1 expression in IL10^low^ tumors (top left), IL10^high^ tumors (top right), PTGS2^low^ tumors (bottom left) and PTGS2^high^ tumors (bottom right) in the SARC STS cohort

The prognostic significance of marker-defined immune cell populations in the Karolinska STS cohort was investigated by Kaplan–Meier survival analysis (Table 3; Fig. 2a). The presence of CD20-positive lymphocytes was associated with better MFS (*P* = 0.009) and OS (*P* = 0.022). Using Cox regression analysis, a significant association between CD20 B cell-positive tumors and improved patient MFS (*P* = 0.021) and OS (*P* = 0.037) remained. The use of CD20 as an independent prognostic marker was further supported by a Spearman correlation analysis demonstrating that CD20 expression did not correlate with previously reported prognostic factors such as volume (*P* = 0.542), grade (*P* = 0.489) and necrosis (*P* = 0.803), or intravascular growth (*P* = 0.232). Among these factors, only tumor grade was associated with worse MFS (*P* = 0.044) and OS (*P* = 0.016) by Kaplan–Meier survival analysis (Supplementary Table 1). Tumor grade is included in the current staging system of STS, and the multivariate Cox regression analysis supported that the grade and presence of CD20-positive lymphocytes act as independent prognostic markers (*P* < 0.05).

**Table 3:** CD20-positive cells in whole tissue sections are prognostic for patient survival

Immune cell marker	IHC score	Metastasis-free survival		Overall survival	
		Survival in months (95% CI)	*P* (log-rank)	Survival in months (95% CI)	*P* (log rank)
Macrophages					
CD68	Low	30 (7–54)	0.532	32 (10–53)	0.349
	High	42 (30–53)		45 (35–57)	
CD80	Low	37 (25–48)	0.228	41 (31–52)	0.268
	High	50 (29–67)		48 (30–67)	
CD163	Low	36 (24–48)	0.226	41 (30–52)	0.302
	High	53 (35–70)		53 (36–70)	
B cells					
CD20	Low	29 (16–41)	*0.009* ^†^	34 (22–45)	*0.022* ^‡^
	High	61 (50–72)		60 (51–69)	
PAX5	Low	37 (25–50)	0.497	42 (30–54)	0.596
	High	45 (28–62)		47 (32–61)	
CD19	Low	42 (31–53)	0.576	46 (36–55)	0.424
	High	28 (0–57)		28 (0–57)	
T cells					
CD8	Low	35 (18–51)	0.256	38 (21–54)	0.347
	High	44 (31–57)		47 (36–58)	
FOXP3	Low	41 (29–53)	0.891	45 (34–56)	0.979
	High	33 (17–49)		35 (20–50)	

Kaplan–Meier survival analysis using the log-rank test for comparison between low versus high IHC score of tissue sections stained for listed immune cell markers in the Karolinska STS cohort. Survival curves with *P* < 0.05 are presented in Fig. 2a

^†^HR = 0.215 (0.058–0.789), *P* = 0.021 in multivariate Cox regression

^‡^HR = 0.282 (0.086–0.926), *P* = 0.037 in multivariate Cox regression

### MS4A1 expression is prognostic for overall survival

To validate the findings on CD20 in a larger STS cohort, the publicly available SARC TCGA dataset was used. This approach confirmed that high and moderate RNA expression of the MS4A1 gene, coding for the CD20 protein, was strongly associated with a favorable prognosis (OS, adjusted *P* value = 0.002) (Fig. 2b). A similar gene analysis was made for CD19, which is another established B cell marker (OS, adjusted *P* value = 0.112) (Fig. 2c). However, the gene expression levels for CD19 were notably low and, taken together, the results indicated that MS4A1 was the preferred B cell marker to use in the prognostic setting.

### MS4A1/CD20 positivity is indicative of B cell differentiation and T cell activation

The MS4A1 gene correlation analysis, with CD27 as the listed top gene (Spearman correlation), indicated a biological role of CD20-positive B cells in antigen presentation, immune cell differentiation and T cell activation (Table 4). Therefore, the analysis of prognostic CD20-positive B cells was continued by multiplex IHC to identify interacting lymphocyte subsets. Cellular clustering of immune cells of different phenotypes was frequently observed. In tumors with CD20-positive B cell infiltration, these cells were seen juxtaposed to CD8-positive T cells in 9 out of 12 cases (Supplementary Fig. 2a). By immunofluorescence, interactions with FOXP3-positive cells were also observed (Supplementary Fig. 2b). This latter approach clearly demonstrated that in all investigated tumors, none of the detected B cells was positive for FOXP3.

**Table 4:** MS4A1 gene expression correlates with genes involved in immune cell function

Top genes*	Gene correlations with MS4A1	
	Spearman	Pearson
CD27	*0.77*	0.51
CD5	*0.76*	0.66
CXCR3	*0.76*	0.45
ADAM6	*0.76*	0.60
DCANP1	*0.76*	0.49
FCRL2	0.64	*0.99*
CD19	0.54	*0.99*
VPREB3	0.41	*0.98*
FAM129C	0.48	*0.98*
PAX5	0.47	*0.98*

Spearman and Pearson correlation analysis obtained via the cBioportal interactive tool and the study on STS (TCGA, provisional) (http://www.cbioportal.org/)

*First five genes ranked according to Spearman, second five according to Pearson (in italics)

### An immunosuppressive tumor microenvironment attenuates the prognostic impact of MS4A1

To further explore the B cell phenotype in STS, IL10 expression was specifically analyzed. High levels of IL10 in tumors are generally considered to reflect an immunosuppressive tumor microenvironment, which our presented data on CD163 expression and M2 macrophage polarization supported. IL10-producing B cells, often called regulatory B cells, have also been described, and these cells may regulate immune responses in a suppressive manner [27]. Notably, the prognostic role of MS4A1 expression was only observed in tumors with low IL10 expression (adjusted *P* value = 0.002) and not in tumors with high IL10 expression (adjusted *P* value = 0.211) (Fig. 2d top). A similar effect was observed for expression of the gene coding for COX-2, PTGS2 (adjusted *P* value = 0.003 in low expressing tumors versus adjusted *P* value = 0.210 in high expressing tumors) (Fig. 2d bottom), as well as for CD163, even though less pronounced (adjusted *P* value = 0.04 in low expressing tumors versus adjusted *P* value = 0.06 in high expressing tumors; Supplementary Fig. 3). A gene coexpression summary of all the investigated M1/M2 macrophage markers analyzed in the SARC STS cohort further showed that IL10 was often co-expressed with CD163 (Supplementary Table 2). Altogether, these data indicated that the observed prognostic impact of MS4A1 expression is altered by the immune profile of the tumor microenvironment.

## Discussion

Immune cells have been poorly documented in STS as opposed to other more common malignancies. This is one of the first tumor microenvironmental studies systematically investigating the expression and abundance of different leukocyte markers, associated cytokine profiles and cellular interaction partners, and finally, the prognostic outcome in terms of patient survival analyzed in two independent patient cohorts.

Infiltration of M2-polarized macrophages was a rather ubiquitous phenomenon in all investigated tumors, and consequently not associated with prognosis. However, this observation supports the idea of therapeutic intervention, where tumor-associated macrophages could be a potential target. Notably, the alkylating agent trabectedin, which obtained initial US approval in 2015 for treatment of a subset of patients with advanced STS [28, 29], presumably targets tumor-associated macrophages as was recently demonstrated in several different mouse models [16].

In contrast to the highly abundant macrophages, B cells were absent in many investigated tumor areas. The prognostic role of CD20-positive B cells was however noteworthy. Our findings support previous work [30] and further show that the observed correlation with improved patient survival can be detected both at the RNA level and by IHC in tissue sections. For a prognostic marker, this is clearly an advantage and creates flexibility in the clinical setting. However, the prognostic value of immunostaining for CD20 was only evident when a combined IHC score from three tissue sections from the same tumor sample was applied. This indicates that IHC analysis of low-abundant cell types, such as CD20-positive B cells, should be carried out in whole tissue sections, rather than in tissue cores, to reach maximal sensitivity.

The prognostic significance of other B cell markers in the Karolinska STS cohort was not observed when the tissue specimens were analyzed by IHC. The CD19 protein was almost undetectable, suggesting that B cells to a large extent lose their CD19 expression during cellular maturation. This would then be in line with the clinical outcome being related to effector B lymphocytes rather than precursor cells. Still, Pearson’s correlation analysis demonstrated a strong correlation between MS4A1 and CD19 on the RNA level in the SARC STS cohort. A similar correlation was also noted with the nuclear antigen PAX5, which is another commonly used B cell marker. Most CD20-positive cells co-expressed PAX5 in the Karolinska STS cohort, but there was not a complete overlap and, altogether, the data suggested that CD20 was the preferred marker for prognostic B cells.

Although the molecular pathways driving B cell function in STS remain to be characterized, our data rule out the possibility of a B regulatory cell phenotype [31]. A gene correlation analysis suggested a biological role of MS4A1/CD20-positive cells in antigen presentation, immune cell differentiation and T cell activation, further supported by multiplex IHC demonstrating that these cells interacted with both CD8- and FOXP3-positive cells in tumor tissue. However, to what extent the listed genes that correlated with MS4A1 are involved in B cell function in the tumor microenvironment remains to be explored. The cell surface marker CD5 can for example be expressed on both T and B cells, and the need for direct cell–cell contact provides an additional level of complexity for co-stimulatory molecules such as CD27 [32–36].

Irrespective of the mechanism, B lymphocyte function appeared to be attenuated in a tumor microenvironment rich in IL-10 or PTGS2, coding for COX-2, or to a lesser extent also CD163. Both IL-10 and COX-2 are associated with pleiotropic activities in tumor biology and macrophage plasticity. M2-like macrophages are in general major producers of IL-10, which was also supported by our data showing a strong correlation between IL-10 and the tumor-associated macrophage marker CD163 in both patient cohorts. Clearly, it would be interesting to further explore how macrophage-targeting agents like trabectedin alter the tumor microenvironmental profile of STS, and possibly also B cell function.

To conclude, the identification of CD20/MS4A1 as a biomarker for improved patient survival in multiple cohorts is promising and implies that further clinical development of this molecular tool should be considered. However, our results also indicate that the tumor microenvironmental immune profile, defined by IL10 or PTGS2 gene expression levels, seems to alter the prognostic potential of CD20/MS4A1. The presence of macrophages strongly correlated with IL-10 expression, indicating that they are a major source of IL-10 production. Accumulating evidence hereby suggest that M2-polarized tumor-associated macrophages represent a promising target for STS immunotherapy.

## Electronic supplementary material

Below is the link to the electronic supplementary material.

Supplementary material 1 (PDF 38613 KB)

## Abbreviations

M1: Type 1 macrophages
M2: Type 2 macrophages
MFS: Metastasis-free survival
NOS: Nitric oxide synthase
OS: Overall survival
PTGS2: Prostaglandin-endoperoxide synthase 2
REMARK: Reporting recommendations for tumor marker prognostic studies
STS: Soft tissue sarcoma
SARC: Sarcoma Alliance for Research through Collaboration
TCGA: The Cancer Genome Atlas
