# Supplementary material for: An immunosuppressive macrophage profile attenuates the prognostic impact of CD20-positive B cells in human soft tissue sarcoma
Source: Cancer Immunol Immunother. 2019 Mar 16;68(6):927–36. doi: 10.1007/s00262-019-02322-y (PMC6529392; doi:10.1007/s00262-019-02322-y)
Supplement: Supplementary file 1 — Supplementary material 1 (PDF 38613 KB) [file 262_2019_2322_MOESM1_ESM.pdf]

a

IHC Scoring		CD20	CD19	PAX5
**0**	(negative)	17	27	17
**1**	(1-10 cells*)	6	2	10
**2**	(11-300 cells*)	7	1	3
**3**	(>300 cells*)	0	0	0

*Estimated number of cells/tissue section (0.8 cm^2)

b

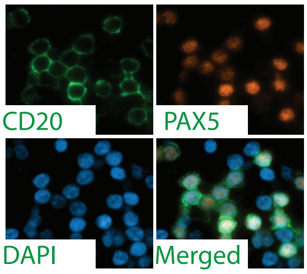

Supplementary Fig. 1 CD20-positive B cells often coexpress PAX5.
a Overview of the IHC scoring for B cell markers in the Karolinska STS cohort. Note: 15 out of the 17 tumors that were scored negative for CD20 were also negative for PAX5. **b** Immunostaining for CD20 and PAX5 in STS tissue sections showing that the vast majority of CD20-positive cells coexpress PAX5.

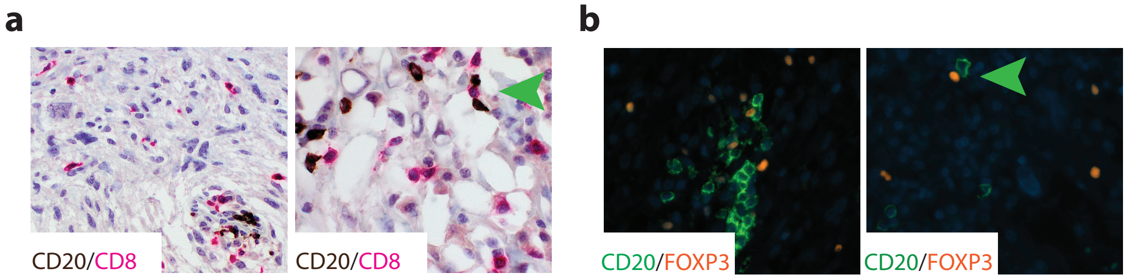

Supplementary Fig. 2 CD20-positive cells interact with CD8-positive cells and FOXP3-positive cells. **a** Multiplex IHC for CD20 (brown) and CD8 (red), Htx as counterstain, demonstrating direct cell-cell interactions. **b** Immunostaining for CD20 (green fluorophore) and FOXP3 (red chromogenic, fluorescent substrate) demonstrating direct cell-cell interactions. Nuclei are visualized by DAPI.

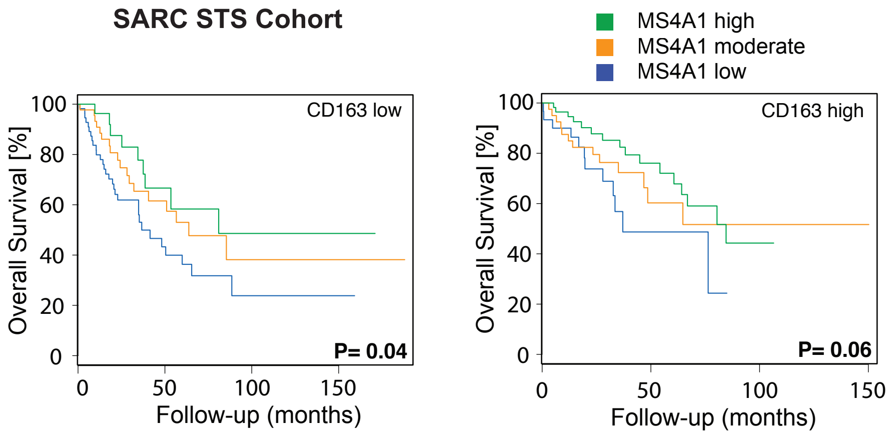

Supplementary Fig. 3 Kaplan−Meier analysis illustrating the prognostic impact of MS4A1 expression in the SARC STS cohort comparing tumors with low and high expression of CD163.

Supplementary Table 1 Prognostic significance of CD20 IHC score in comparison to other previously reported prognostic factors. Kaplan−Meier survival analysis using the log-rank test for comparison between groups in the Karolinska STS cohort. The results remained significant in a multivariate Cox regression analysis (see table foot notes and text for details).

	**Metastasis-free Survival**		**Overall Survival**	
**Prognostic Factor**	**Survival in months (95% C.I.)**	**P (log rank)**	**Survival in months (95% C.I.)**	**P (log rank)**
**CD20**				
low	29 (16-41)	**0.009†**	34 (22-45)	**0.022†**
high	61 (50-72)		60 (51-69)	
**Tumor volume**				
small	50 (37-61)	0.142	50 (37-61)	0.231
large	35 (22-49)		37 (24-51)	
**Tumor grade**				
3	57 (45-70)	**0.044†**	61 (50-72)	**0.016†**
4	39 (28-50)		40 (28-50)	
**Necrosis**				
No	48 (47-49)	0.822	48 (47-49)	0.900
Yes	41 (31-51)		42 (32-52)	
**Intravascular growth**				
No	48 (37-58)	0.730	50 (38-60)	0.637
Yes	36 (15-57)		36 (15-57)	

†P<0.05 in multivariate Cox regression

Supplementary Table 2 Co-occurrence of analyzed M1/M2 macrophage gene expression markers in the SARC STS cohort. A gene coexpression analysis demonstrates a correlation between the M1 macrophage markers NOS2 and NOS3, and a correlation between the M2 macrophage markers CD163 and IL10, using the cBioportal software analysis tool.

Gene A	Gene B	Neither	A Not B	B Not A	Both	Log Odds Ratio	P Value	Adjusted*
NOS2	NOS3	251	3	6	3	>3 (co-occurance)	**<0.001**	**0.005**
IL10	CD163	242	8	9	4	2.599 (co-occurance)	**0.001**	**0.014**
IL10	PTGS2	238	12	13	0	<-3	0.537	1.000
PTGS2	CD163	237	13	13	0	<-3	0.509	1.000
IL10	NOS2	245	12	6	0	<-3	0.754	1.000
PTGS2	NOS2	244	13	6	0	<-3	0.736	1.000
CD163	NOS2	244	13	6	0	<-3	0.736	1.000
IL10	NOS3	242	12	9	0	<-3	0.652	1.000
PTGS2	NOS3	242	12	8	1	0.925	0.371	1.000
CD163	NOS3	241	13	9	0	<-3	0.629	1.000

*Bonferroni adjusted P Value.
